# The Growth Kinetic and Ultra High Hardness of CoCrFeNiTi High–Entropy Alloy by Mechanical Alloying and Spark Plasma Sintering

**DOI:** 10.3390/ma18143242

**Published:** 2025-07-09

**Authors:** Tiejun Qu, Mingpu Liu, Chuanhua Yang, Xin Wang, Junfa Wang

**Affiliations:** 1 School of Materials Science and Engineering, Jiamusi University, Jiamusi 154007, China; 2 School of Materials Science and Chemical Engineering, Harbin University of Science and Technology, Harbin 150080, China

**Keywords:** high entropy alloy, CoCrFeNiTi, mechanical alloying, spark plasma sintering, growth kinetic

## Abstract

In this paper, the impact of mechanical alloying (MA) and spark plasma sintering (SPS) on the phase evolution and mechanical properties development of CoCrFeNiTi high–entropy alloys (HEAs) was investigated. The microstructure and properties of the material were examined, using X-ray diffraction (XRD) for phase identification, scanning electron microscopy (SEM) for surface morphology observation, transmission electron microscopy (TEM) for microstructural analysis, and hardness testing to evaluate mechanical performance. The milled powder exhibited nanocrystalline solid solution microstructure with grain sizes below 48 nm, composed of 83% face–centered cubic (FCC) and 17% body–centered cubic (BCC) phases. Mechanically, the bulk CoCrFeNiTi alloy exhibited exceptional strength attributes, as evidenced by a Vickers hardness value reaching 675 Hv, along with a compressive strength of 1894 MPa and a yield stress of 1238 MPa. These findings suggested that the synergistic effects of mechanical alloying and SPS processing can precisely control the phase stability, microstructure refinement, and property optimization in CoCrFeNiTi HEA, with particular promise for advanced structural applications.

## 1. Introduction

High–entropy alloys (HEAs) have had remarkable progress and advancement since their development by Professor Yeh in 2004. The term “high–entropy alloy” generally refers to a single–phase solid solution alloy with a simple crystal structure composed of five or more elements, with an atomic percentage ranging from 5% to 35% [1,2,3]. Given their superior hardness, exceptional strength, resistance to corrosion, and a multitude of additional benefits, HEAs have became a new type of alloy material that has received a great deal of attention in the research field during recent years and the hot point of many researches [4,5,6,7]. The high–entropy alloy composed of five elements–Cobalt (Co), Chromium (Cr), Iron (Fe), Nickel (Ni), and Titanium (Ti)–in equimolar or nearly equimolar proportions exhibited a simple solid solution structure and favorable plasticity and machinability. However, it suffered from a relatively low hardness and strength [8,9,10]. The existing research lacked a comprehensive and thorough understanding of the microstructure and deformation dynamics of the CoCrFeNiTi high–entropy alloy.

High–entropy alloys (HEAs) exhibit remarkable properties, such as exceptional strength coupled with ductility, elevated strength at high temperatures, thermal stability, corrosion resistance, and notable electrical resistance [11,12,13,14]. There are four ways to fabricate HEAs: melting, gas–phase synthesis, electrochemical techniques, and solid–state processing. Among these, casting worked as the predominant method employed for HEA production. However, the casting process is fraught with challenges [15,16,17,18]. During solidification, thermal expansion and contraction occur, giving rise to numerous structural imperfections, notably porosity. Consequently, the microstructure resulting from solidification is far from homogeneous [19]. This non–uniformity in the microstructure can subsequently lead to heterogeneous material properties, which may compromise the alloy’s performance in certain applications. Mechanical alloying, a solid–phase manufacturing technique, facilitates the production of high–entropy alloys (HEAs) with homogeneity superior to that of the casting method. Given its efficacy in generating solid solutions, mechanical alloying enables the synthesis of nanostructured HEAs [20,21]. Spark Plasma Sintering (SPS), a technique that uses electric current to trigger the consolidation and reaction sintering processes of materials, offers distinct benefits when compared to time–consuming conventional methods such as hot pressing, hot isostatic sintering, high–frequency induction heating, isostatic pressing and sintering (a combination process), hot isostatic pressing (the repeated mention here is for clarity in contrasting with SPS), and liquid phase sintering. It enables substantial material compaction at relatively low temperatures and within a shorter timeframe [22,23,24]. Zeraati M [25] reported an AlCrCuFeNiTi HEA synthesized through MA, followed by consolidation using SPS. The mechanical properties of the consolidated sample were remarkable, featuring a yield stress of 2004 MPa, a compressive strength of 2811 MPa, and a Vickers hardness of 684 HV. Adabavazeh, Z [26] focused on the fabrication and assessment of a CuFeNiMnTi HEA, synthesized via MA and SPS. The sintered CuFeNiMnTi alloy exhibited a fracture toughness of 9.24 ± 0.5 MPa root at room temperature, along with significant mechanical enhancements, including a microhardness of 563 ± 22 HVN and an Ultimate Shear Strength (USS) of 439 ± 19 MPa. Yurchenko, N [27] reported low–density AlCrFeTiX (where X = Co, Ni, Cu) high–entropy alloys made using MA and SPS. Compression testing revealed that the SPS–processed AlCrFeTiX (X = Co, Ni, Cu) alloys exhibited brittle behavior at room temperature (25 °C). At an elevated temperature of 600 °C, the SPS–processed AlCrFeTiCo alloy exhibited a yield strength of 1960 MPa, a peak strength of 2121 MPa, and a plastic strain of 1.8%. These alloys exhibited remarkable properties, including elevated hardness and outstanding compressive performance.

A number of studies have explored the synthesis of HEAs with compositions similar to the alloy under investigation in this study. Notably, while many of these studies have focused on single–phase HEAs, the combination of body–centered cubic (BCC) and face–centered cubic (FCC) phases had shown superior properties [28]. High–entropy alloys (HEAs) with body–centered cubic (BCC) structures are renowned for their remarkable strength, yet they lack the flexibility inherent in other alloy types [20,29]. In stark contrast, HEAs exhibiting face–centered cubic (FCC) structures, while weaker in terms of strength, offer superior flexibility. For achieving the balanced combination of elevated strength and flexibility, alloys often require a dual–phase microstructure integrating face–centered cubic (FCC) and body–centered cubic (BCC) phases. The development of a solid solution within these alloys typically enhances their overall mechanical properties, making them more robust and adaptable for various applications [30,31]. The kinetics of alloys have not been thoroughly examined in numerous prior investigations. In particular, the mechanical properties of the CoCrFeNiTi alloy have remained largely unexplored.

In this paper, our work focuses on the unique combination of elements and the synthesis approach employed. By utilizing mechanical alloying followed by SPS, we were able to achieve a fine–grained microstructure in CoCrFeNiTi alloys, which contributed to their exceptional mechanical properties. Furthermore, our study also focused on the kinetics analysis, which was crucial to the experimental findings.

## 2. Materials and Methods

High–entropy alloy powders composed of CoCrFeNiTi were prepared using a mechanical alloying process through high–energy ball milling (HEBM) under argon atmosphere. These elemental powders included cobalt (99.8% purity, particle ranging from 45 µm to 60 µm), chromium (99.7% purity, particle ranging from 10 µm to 30 µm), iron (99.96% purity, particle ranging from 10 µm to 20 µm), nickel (99.5% purity, particle ranging from 45 µm to 60 µm), and titanium (99.95% purity, particle ranging from 2 µm to 5 µm). The milling process was conducted using a planetary ball mill model QM–1SP4, utilizing a stainless steel cylindrical vessel and balls with diameters between 5 mm and 10 mm. A ball to mill weight ratio of 20:1 was maintained. The milling container was evacuated and filled with argon gas three times to ensure an inert atmosphere. The ball milling was executed at a rotational speed of 400 rpm, with milling durations spanning from 1 h to 6 h.

**Figure 1:**
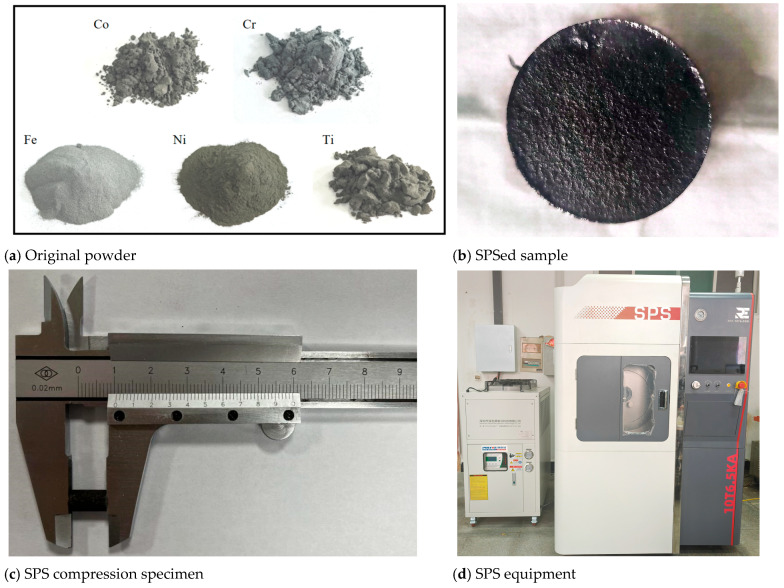
The representation images of power, SPSed sample, and equipment.

The bulk CoCrFeNiTi high–entropy alloy was prepared via spark plasma sintering (SPS) in a vacuum environment. The powder was placed in a cylindrical graphite mold with a 30.4 mm internal diameter. The heating process was initiated at a rising rate of 50 °C/min until reaching the sintering temperature in the range of 900 °C to 1000 °C. The samples remained at the sintering temperature for 10 min. Figure 1 presents the original powder utilized in this study, the SPS–processed sample, the SPS compression specimen, and the SPS equipment. Notably, the disk prepared via SPS measured 10 mm in thickness and 6 mm in diameter.

To study phase transformation, X-ray diffraction (XRD) analysis was used, equipped with Cu–Kα radiation (λ = 0.15406 nm). The diffraction patterns were recorded with a 2θ range of 20°to 80° and diffractometer step of 0.05°. For structural characterization, scanning electron microscopy (SEM, HITACHI, SU8010, Tokyo, Japan) euqipped with energy dispersive spectroscopy (EDS) was conducted using a Nanosem 450 microscope. The Vickers hardness measurements were taken at ten distinct locations on each sample to calculate the mean hardness value. The sintered alloy samples were ultrasonically cleaned in ethanol for 10 min to remove any surface contaminants. Indentations were made at a load of 0.05 kN with a dwell time of 15 s. Compressive strength tests were conducted on five samples, and the average values were reported for analysis. The specimens were loaded at a constant displacement rate of 0.5 mm/min, corresponding to an initial strain rate of 1 × 10^−3^ s^−1^.

## 3. Results

### 3.1. Phase Prediction

To predict the formation of a solid solution in the CoCrFeNiTi system, the Hume–Rothery rule, a widely recognized and established standard, was employed. The atomic radii (∆r), electronegativity (∆χ) and the average valence electron concentration (VEC) were sourced from Table 1.

In the CoCrFeNiTi high–entropy alloy, the mixing entropy can be calculated by:

(1) $${∆S}_{mix}=-R\sum_{i=1}^{n}c_{i}lnc_{i}$$

where R is the gas constant and c_i_ is the mole fraction of constituents i.

The formation of intermetallic compounds can be evaluated based on valence electron concentration (VEC), defined by Equation (2).

(2) $$VEC=\sum_{i=1}^{n}{\left(VEC\right)}_{i}$$

VECi corresponds to the VEC value for element. It represents the average mobile electron number for every atom. Additionally, the influence of valence electron concentration (VEC) on the phase stability between the amorphous and solid solution phases can be ignored. However, Gio et al. [1] highlighted that VEC is crucial in dictating the formation of solid solutions in high–entropy alloys (HEAs), with the FCC structure forming when the VEC was 8 or higher, and the BCC structure emerging when the VEC was below 6.87.

Additionally, Δ*H*_mix_ is defined by Equation (3).

(3) $${∆H}_{\mathrm{mix}}=\sum_{i=1,i\neq j}^{n}\Omega_{ij}C_{i}C_{j}$$

(4) $$\Omega_{ij}={∆}_{mic}^{AB}$$

$\Delta_{mic}^{AB}$ is the mixing enthalpy for AB binary alloys, determined through the application of the Miedema model in Table 1.

The CoCrFeNiTi alloy’s thermodynamic parameters, which incorporated the enthalpy value, are subsequently presented in Table 2.

The mixing enthalpy (ΔHmix) and mixing entropy (ΔSmix) for CoCrFeNiTi were approximately –4.08 kJ/mol and 15.53 J/(K·mol), respectively. Additionally, the atomic size difference (δ) for the CoCrFeNiTi alloy was 6.15, which lies within the range of 0 ≤ δ ≤ 8.5. Furthermore, the valence electron concentration (VEC) was 7.2, falling within the range of 6.87 ≤ VEC ≤ 8, suggesting the phase forming of both face–centered cubic (FCC) and body–centered cubic (BCC) structures. Collectively, these thermodynamic parameters predicted the emergence of solid solution structures encompassing both FCC and BCC.

### 3.2. Microstructural and Phase Evolution

**Figure 2:**
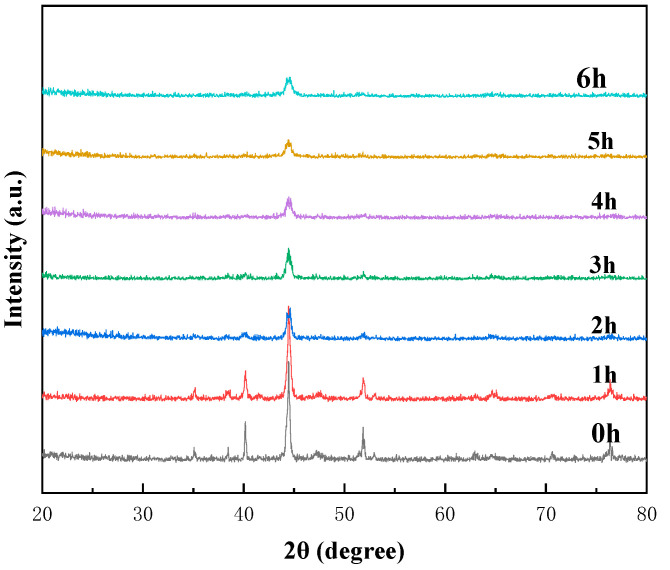
XRD pattern of CoCrFeNiTi high–entropy alloy were milled for 0 h–6 h.

Figure 2 presents the X-ray diffraction (XRD) patterns of the CoCrFeNiTi high–entropy alloy powder subjected to mechanical milling at room temperature for different times: 0 h, 1 h, 2 h, 3 h, 4 h, 5 h, and 6 h. Initially, at 0 h of ball milling, the XRD spectrum displayed peaks corresponding to all of the constituent elements. These peaks persisted even after 1 h of milling. However, as the milling time reached 2 h, a discernible decrease in peak intensity and an increase in peak width were observed, attributable to internal strains and the refinement of crystal sizes within the powder particles. Additionally, a reduction in peak heights accompanied by shifts in their positions indicated the formation of a solid solution involving the initial elements.

After 3 h of mechanical alloying, the XRD pattern (Figure 1) highlighted the emergence of two peaks at 2θ angles of approximately 40° and 56°, corresponding to the hexagonal close–packed (HCP) structure. This phase formation was likely linked to the presence of Ti, which has a relatively high melting point and the largest atomic radius among the elements, facilitating the formation of an HCP crystalline lattice.

Following 4 h of mechanical alloying, the original elements of the high–entropy alloy gradually dissolved, leading to the formation of the solid solution. This transition was marked by the disappearance of diffraction peaks corresponding to the pure elements. The solid solution exhibited a dual–phase structure, including both face–centered cubic (FCC) with lattice planes (111), (200), and (222), and body–centered cubic (BCC) with lattice planes (110) and (200). These two crystalline phases remained stable within the solid solution until the milling duration reached 6 h. The structural changing observed during ball milling can be substantiated through thermodynamic analyses.

During the milling process, the XRD peaks exhibited a broadening in width and reductions in intensity attributable to the refinement of the powder particles and the increasing of internal strain. Consequently, both the lattice strain and the crystal size were influenced by milling time. According to the Williamson–Hall relationship, prolonged milling reduces crystal size while simultaneously increasing lattice strain. Table 3 showed the crystal size of CoCrFeNiTi high–entropy alloy milled with different time.

### 3.3. SEM Results

**Figure 3:**
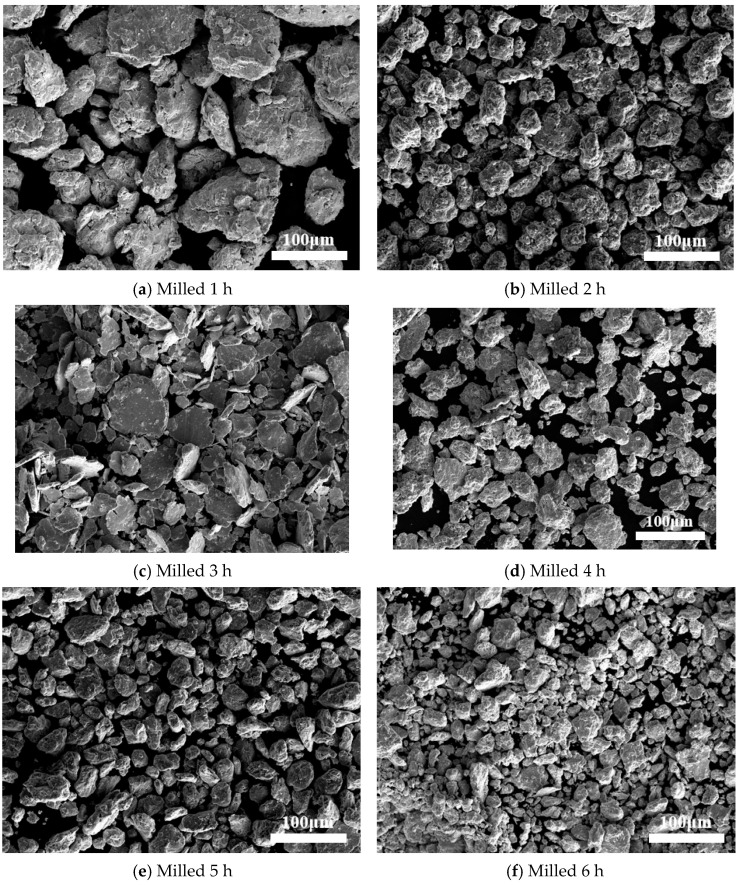
SEM images related to (**a**) 1 h, (**b**) 2 h, (**c**) 3 h, (**d**) 4 h, (**e**) 5 h, and (**f**) 6 h of ball milling.

Figure 3 presented the secondary field–emission scanning electron microscopy (FE–SEM) images of powder samples followed by milling holding time of 1 h, 2 h, 3 h, 4 h, 5 h, and 6 h. For the 1 h milled alloy sample (Figure 3a), the presence of round and raised surfaces indicated the agglomeration of powders with an average particle size of approximately 100 ± 5 µm. Figure 3b illustrates the powder particles after 2 h of milling. Notably, the initially spherical particles were no longer discernible in samples milled for 2 h. The average particle size decreased to about 60 ± 5 µm, signifying structural distortions due to compaction. SEM images of the 5 h MA sample are depicted in Figure 3e. Smaller and dispersed powder particles which were finer than those observed in the 1 h, 2 h, 3 h, and 4 h milled samples were evident. The surfaces of these fine particles exhibited no agglomeration signs. Extending the milling time to 6 h (Figure 3f) led to further particle crushing and yielded even finer particles, reducing the average size to approximately 30 ± 5 µm. The intense collisions during the process induced significant cold working and work hardening effects in the particles, leading to a decrease in the average particle size, as clearly illustrated in Figure 3f.

The diminishing average particle size over prolonged milling periods underscores the dominance of the fracture process over cold welding. The broader peaks observed in the X-ray diffraction patterns of powder mixtures subjected to extended milling times (Figure 2) provide further evidence of severe cold deformation within the powder particles. Following prolonged milling, the powder particles achieved a high degree of homogeneity in both size and shape, as well as in their constituent elements and compositions. The mechanical alloying process facilitated the mutual dissolution of the initial elements, leading to the formation of supersaturated solid solutions. This achievement represents a significant milestone in the development of novel alloys and engineering materials with tailored properties.

**Figure 4:**
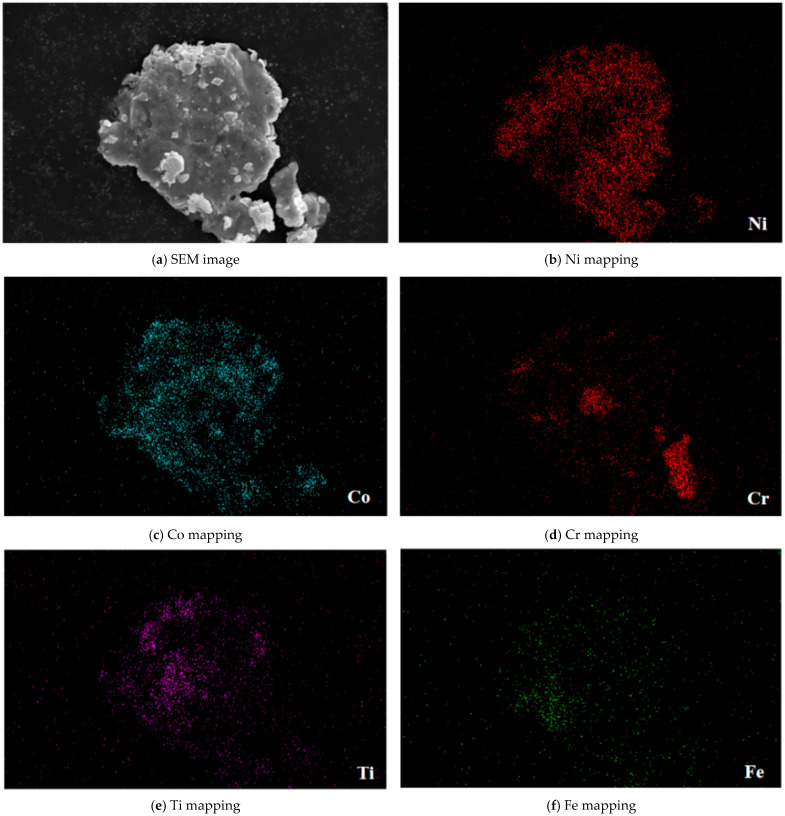
SEM image and elemental distribution map scanning for 6 h.

Figure 4 presents the EDS pattern of CoCrFeNiTi powder followed by mechanical alloying for 6 h. The results indicate a higher proportion of iron in the powder. Figure 4b–f depicts the corresponding EDS MAP analysis. According to this analysis, elements such as Fe, Ti, and Cr exhibited a uniform distribution, whereas Co and Ni displayed a distinct distribution pattern. The two distinct distribution patterns observed in the final sample correspond to the FCC and BCC phases, respectively. Due to their intrinsic crystal structures, elements such as Fe, Cr, and Ti exhibited a tendency to form solid solutions within the BCC phase, whereas Co and Ni were more inclined to form solid solutions within the FCC phase. The chemical homogeneity was assessed using Energy Dispersive Spectroscopy (EDS), as depicted in Figure 3a–d. The EDS analysis confirmed the uniform distribution of all elements across the sample.

**Figure 5:**
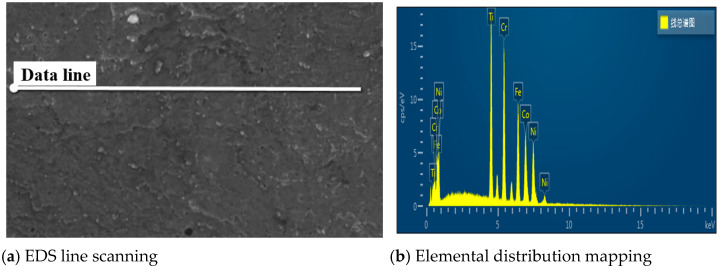
(**a**) EDS Line Scanning (**b**) Corresponding Elemental Distribution Map. The Chinese characters in the figure are “EDS Line—Scan Total Spectrum”.

The EDS line scanning image, as shown in Figure 5, presents a linear path across the sample surface, capturing subtle variations in material composition. The corresponding elemental distribution map illustrates the relative abundance of elements such as Co, Ti, Cr, Ni, and Fe along this path, revealing their spatial distribution patterns. Table 4 presents quantitative data on the spatial distribution of elements detected along the scanned line via EDS analysis.

### 3.4. JMA Kinetic Method Calculations

To comprehensively analyze solid–state reactions, it was essential to employ kinetic models that accounted for both the chemical and geometric–physical characteristics of the system. The reaction kinetics can be categorized into three primary mechanisms: the nucleation and growth mechanism, diffusion–controlled mechanism, and reaction–controlled mechanism. These mechanistic classifications facilitated the development of effective models for studying solid–state reaction kinetics. The models, which are summarized in Table 5, offer insights into the mechanisms governing the reaction’s progress.

The phase fraction can be mathematically represented by Equation (5), including homogeneous nucleation, in which nucleation and growth occur concurrently and heterogeneous (non–homogeneous) nucleation, in which nucleation precedes the growth.

(5) $$\alpha (t)=1-exp(-g\int_{0}^{1}I(t^{\prime }){\left(\int_{t^{\prime }}^{t}U(t\prime \prime )dt\prime \prime \right)}^{3})dt^{\prime }$$

The shape factor, nucleation rate, time, and growth rate are symbolized by g, U, t, and I, respectively. Both the nucleation rate (U) and the growth rate (I) vary; they can be expressed as Equation (6).

(6) $$\alpha (t)=1-exp(-gIU^{3}t^{4}/4)$$

Consequently, if the number of nuclei (N_0_) formed from the new phase, the value of time (t) can be determined using Equation (7).

(7) $$\alpha (t)=1-exp(-gN_{0}U^{3}t^{3})$$

Drawing from Avrami, Equation (7) can be reformulated as Equation (8).

(8) $$\alpha (t)=1-exp(-kt{)}^{n}$$

Furthermore, this relationship can also be represented by a logarithm, as reformulated in Equation (9).

(9) ln(−ln(1−α(t)))=nln(k)+nln(t)

In the given equation, the Avrami exponent (denoted as n) and the reaction rate constant (denoted as k) were time–independent. Through the determination of the Avrami exponent, it can be concluded that the phase transformation belongs to the nucleation and growth mechanism, which can be derived from the n and k values. Table 6 presents various values of n corresponding to different nucleation types and geometries. This method was subsequently applied to examine the kinetic behavior of the CoCrFeNiTi HEAs.

The formation fraction of FCC and BCC phase was estimated using the subsequent formula:

(10) $$\alpha (t)=I_{t}/I_{60}$$

**Figure 6:**
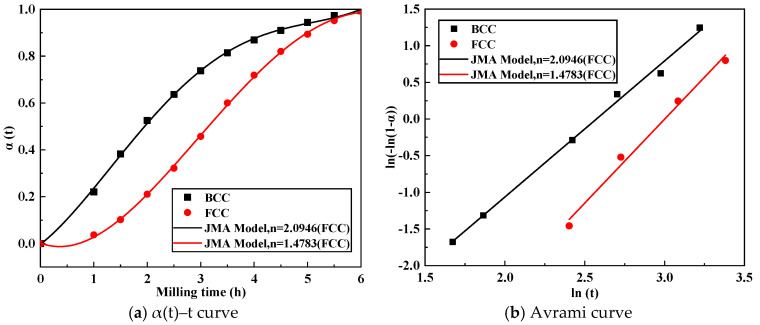
The α(t)–t curve and Avrami curve for CoCrFeNiTi HEA milled at different times.

I_60_ represents the peak intensity of the FCC or BCC phase after 6 h of milling. It represents the peak intensity of the FCC or BCC phase milled at different periods. The angles utilized to identify these phases were 43.4° for FCC and 44.5° for BCC, respectively. Consequently, Figure 6 illustrates the relationship between FCC and BCC phase fraction formation and the milling time. The α(t) plot indicates an approximate nucleation time of about 5 h for the FCC solid solution. Conversely, for the BCC phase, there was no obvious nucleation time, suggesting that the nucleation and growth happened simultaneously with the milling process. Figure 6a reveals that the BCC phase formation rate was higher than the FCC phase formation at the beginning of milling process. However, in the final stages, the BCC formation rate slowed down and the FCC formation rate accelerated. The resulting curve exhibited a nucleation and growth dynamics mechanism during the synthesis procedure. The nucleation of the FCC phase occurred when the ball milling time reached 5 h; then nucleation and growth proceeded simultaneously. In the case of the face–centered cubic (FCC) solid solution phase, nucleation predominantly occurred within the first 5 h of milling, after which nucleation and growth processes proceeded concurrently. Conversely, the BCC phase nucleation and growth occurred from the beginning. Figure 6b presents the Avrami relationship for CuCrFeNiTi, denoting the transformation kinetics for both the FCC and BCC phases.

The Avrami exponent (n) indicates that the formation kinetics of the CuCrFeTiNi high–entropy alloy (HEA) are primarily governed by interface–controlled growth mechanisms. The rate constant (k) for the FCC phase was 0.0305/min, while for the BCC phase it was 0.0412/min. Based on these findings, the kinetic equation for the CuCrFeTiNi alloy can be established as follows:

(11) $${\alpha (t)}_{BCC}=1-exp[-{\left(0.0412t\right)}^{1.48}]$$

(12) $${\alpha (t)}_{FCC}=1-exp[-{\left(0.0305t\right)}^{2.09}]$$

### 3.5. Mechanical Properties

**Figure 7:**
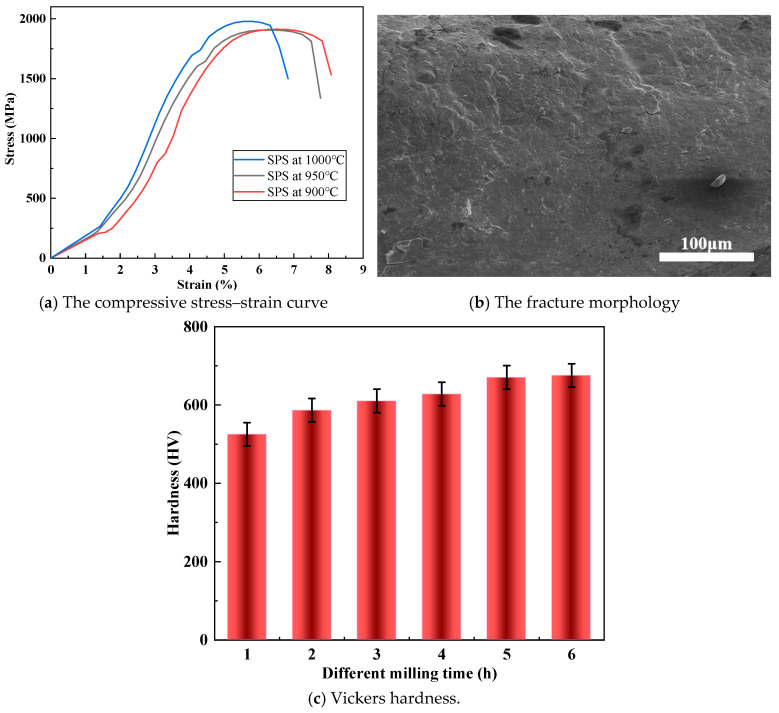
The compressive stress–strain curve, fracture morphology, and Vickers hardness of bulk of CoCrFeNiTi HEA at room temperature.

The bulk CoCrFeNiTi high–entropy alloy exhibited superior microhardness compared to commercial alloys like Stellite (500 Hv) and nickel/cobalt–based superalloys. The enhanced hardness can be attributed to the high titanium content, which induced lattice expansion. Figure 7a displayed the compressive stress–strain curve of the HEAs synthesized at 900 °C, 950 °C, and 1000 °C. The stress–strain curves revealed a clear temperature dependency in the mechanical behavior of the SPS CoCrFeNiTi HEA. The peak stress decreased with increasing temperature. The SPS CoCrFeNiTi HEA tested at 1000 °C exhibited the highest peak stress, followed by 950 °C and 900 °C. The curve indicated that the alloy achieved maximum strength of 1894 MPa and yield stress of 1238 MPa at 1000 °C. Notably, the CoCrFeNiTi high–entropy alloy comprised five distinct elements with nearly equal atomic proportions but different atomic sizes, which contributed to plastic deformation. Furthermore, the current material exhibited a fine–grained microstructure, similar to that observed in previous high–entropy alloys (HEAs), which enhanced its strength. Figure 7b presents the fracture morphologies of the CoCrFeNiTi high–entropy alloy. The SPS–processed CoCrFeNiTi HEA underwent intergranular fracture. Additionally, the regions of deformation are marked by arrows, which confirms that nanoprecipitates play an important role in high strength and low ductility. Figure 7c illustrates the hardness (HV) of bulk CoCrFeNiTi alloy as a function of milling time, ranging from 1 h to 6 h. The hardness values exhibited an increasing trend according to increasing milling time. Initially, the hardness was approximately 525 HV. By 6 h, the bulk CoCrFeNiTi alloy achieved a remarkable Vickers hardness of 675 Hv. This increasing in hardness resulted from grain refinement and enhanced dislocation density due to prolonged milling. The data highlight the effectiveness of extended milling in improving mechanical properties.

**Figure 8:**
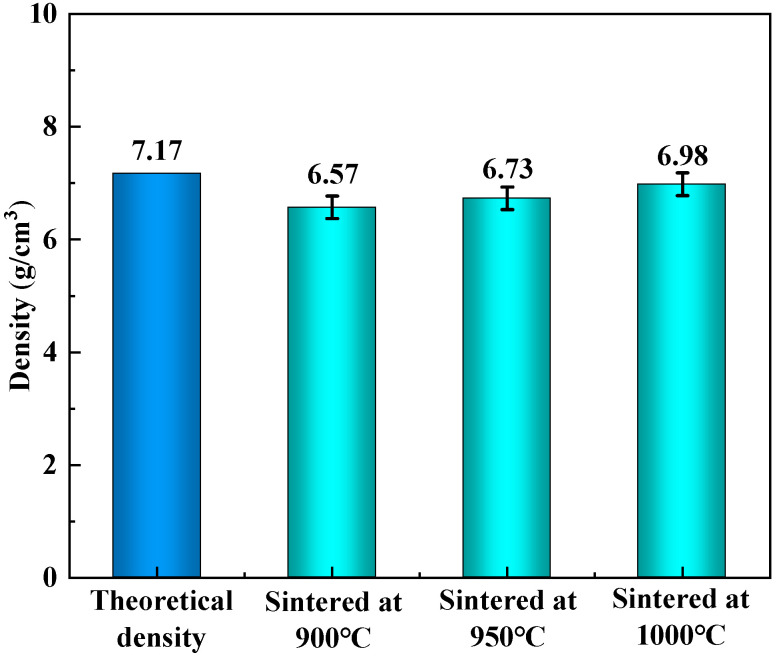
The densities of CoCrFeNiTi HEAs sintered at different temperatures.

Figure 8 shows the measured density of CoCrFeNiTi high–entropy alloy samples produced at different sintering temperatures and compares them with its theoretical density. When the sintering temperature is 950 °C, the measured density of the alloy is much lower than the theoretical density with 6.57 g/cm^3^. Due to the suppression of atomic diffusion at low temperatures, there is a decrease in the interaction between powder particles during the sintering process. The weakening of the bonding strength led to an increase in the number of micropores, ultimately reducing the density of the alloy. As the sintering temperature increases from 1000 °C to 1050 °C, the density of the alloy increases from 6.73 g/cm^3^ to 6.98 g/cm^3^, further approaching the theoretical value.

**Figure 9:**
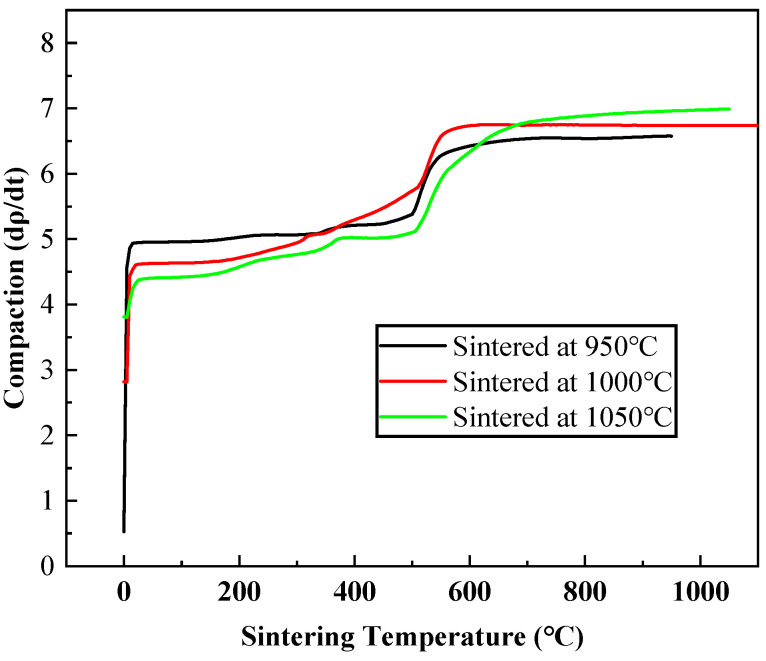
The relationship between the compaction rate (dρ/dt) and sintering temperature.

Figure 9 showed the compaction rate (dρ/dt) as a function of sintering temperature for samples sintered at different temperatures (950 °C, 1000 °C, and 1050 °C). The compaction rate increased with the sintering temperature for all three samples. This was consistent with the fundamental principles of sintering, where higher temperatures provide more energy for particle rearrangement, diffusion, and bonding, thereby accelerating the densification process. At the initial stage of sintering (low–temperature range), the compaction rate was relatively high for all samples. During the initial pressing process the main process was from loose packing to densification, resulting in a fast rate of density change. As the sintering temperature increased in the intermediate range (200–600 °C), the compaction rate started to slow more rapidly. This was because the particles had not yet gained sufficient thermal energy to overcome the barriers to rearrangement and bonding. In the high–temperature stage (above 600 °C), the compaction rate continued to increase, but at a slower pace compared to the intermediate stage. A higher sintering temperature can enhance the compaction rate, but there may be a saturation effect at very high temperatures. This information was crucial to optimizing the sintering process parameters to achieve the desired density and microstructure in the final sintered samples.

## 4. Conclusions

In the present work, the CoCrFeNiTi high–entropy alloy was synthesized by mechanical alloying and SPS. The alloying behavior, structural evolution, and kinetics were analyzed and discussed. Based on the results, the following conclusions were made:

(1) XRD studies showed that the crystal structures of CoCrFeNiTi HEA powders are FCC and BCC phases, which are produced by the mechanical alloying process after ball milling for 6 h. Kinetic investigations, based on the Johnson–Mehl–Avrami (JMA) model, revealed that the formation of the CoCrFeNiTi alloy proceeded through a mechanism of controlled nucleation and growth.

(2) The bulk CoCrFeNiTi high–entropy alloy exhibited a remarkable hardness value of 675 Hv, surpassing that of most commercial alloys. This elevated hardness can be attributed to the refined microstructure, solid solution strengthening effects, and the significant proportion of the BCC phase within the alloy. Furthermore, the bulk CoCrFeNiTi alloy demonstrated a maximum compressive strength of 1894 MPa and a yield stress of 1238 MPa.

## Figures and Tables

**Table 1:** The mixing enthalpy of binary alloys ($\Delta_{mic}^{AB}$) using the Miedema model.

	Fe	Cr	Ti	Ni	Co
**Fe**	-	−1	−17	−2	−1
**Cr**	−1	-	−7	−7	−4
**Ti**	−17	−7	−	−35	−28
**Ni**	−2	−7	−35	-	0
**Co**	−1	−4	−28	0	-

**Table 2:** CoCrFeNiTi thermodynamic parameter.

Parameter	Value
δ	6.15
$\Delta H_{\mathrm{mix}}\;(\mathrm{KJ}/\mathrm{mol})$	−4.08
$\Delta S_{\mathrm{mix}}\;({\mathrm{JK}}^{-1}{\mathrm{mol}}^{-1})$	15.53
VEC	7.2

**Table 3:** The crystal size of the prepared samples.

Sample	Milling Time (h)	Crystal Size (nm)	
		BCC	FCC
**CoCrFeNiTi**	1	108	94
	2	68	74
	3	58	66
	4	38	41
	5	34	38

**Table 4:** The elemental distribution data from EDS line scanning.

Element	Concentration	k Ratio	wt%
**Ti**	46.28	0.46283	20.04
**Cr**	51.33	0.51334	22.64
**Fe**	45.51	0.45513	20.88
**Co**	38.08	0.38085	18.09
**Ni**	39.64	0.39635	18.34

**Table 5:** Functions used for kinetic analysis of solid–state reaction.

	Kinetics Model	Parameter	Plotted Function
Chemical reaction	Maniple	−ln(1−α) vs.t	Slope = kM
	Second–order	$(1-\alpha {)}^{-1}-1\;vs.t$	Slope = kS.O
	Contracting cylinder	1−1−α12 vs.t	Slope = kC.C
	Contracting sphere	1−1−α13 vs.t	Slope = k_C.S_
Diffusion model	Jander	[1−(1−α)13]2 vs.t	Slope = k_j_
	Crank	$1-(2/3)\alpha -(1-\alpha {)}^{2/3}\;vs.t$	Slope = k_C_
	Dunwald–Wagner	$ln(6/[n^{2}(1-\alpha )])\;vs.t$	Slope = k_D,W_
	One–dimensional diffusion	$\alpha^{2}\;vs.t$	Slope = k_O.D.D_
Nucleation–growth	Johnson–Mehl–Avrami	$lnln(\left(1/\left(1-\alpha \left(t\right)\right)\right)\;vs.lnt$	Slope = n, Intercept = nln[k]

**Table 6:** Different nucleus and nucleation geometries and their n values.

	n	Type of Nucleation	Geometries
**Interface–controlled growth**	1	Exhausted rapidly	Plane
	2	Exhausted rapidly	Cylinder
	3	Exhausted rapidly	Sphere
	4	Exhausted rapidly	Sphere
**Diffusion–controlled growth**	1.2	Exhausted rapidly	Plane
	1	Exhausted rapidly	Cylinder
	3.2	Exhausted rapidly	Sphere
	5.2	Exhausted rapidly	Sphere

## Data Availability

The raw data supporting the conclusions of this article will be made available by the authors on request.

## References

[1] Yeh J.-W., Chen S.-K., Lin S.-J., Gan J.-Y., Chin T.-S., Shun T.-T., Tsau C.-H., Chang S.-Y. (2004). Nanostructured high–entropy alloys with multiple principal elements: Novel alloy design concepts and outcomes. Adv. Eng. Mater..

[2] Feng L., Wang G.P., Ma K., Yang W.J., An G.S., Li W.S. (2023). Microstructure and properties of AlCoxCrFeNiCu high entropy alloy coating synthesized by cold spraying assisted induction remelting. Acta Metall. Sin..

[3] He Q.F., Ding Z.Y., Ye Y.F., Yang Y. (2017). Design of High–Entropy Alloy: A Perspective from Nonideal Mixing. JOM.

[4] Li Y.Z., Shi Y. (2021). Microhardness, wear resistance, and corrosion resistance of AlxCrFeCoNiCu high–entropy alloy coatings on aluminum by laser cladding. Opt. Laser Technol..

[5] Wei X., Zhang L., Zhang F., Zhang C., Jia Q., Sun K., Duan D., Jiang H., Li G. (2024). Effect of carbon addition on the microstructure and corrosion resistance of the CoCrFeNi high–entropy alloy. Corros. Sci..

[6] Arif Z.U., Khalid M.Y., Rehman E.U., Ullah S., Atif M., Tariq A. (2021). A review on laser cladding of high–entropy alloys, their recent trends and potential applications. J. Manuf. Process..

[7] Cantor B., Chang I.T.H., Knight P., Vincent A.J.B. (2004). Microstructural development in equiatomic multicomponent alloys. Mater. Sci. Eng. A.

[8] Otto F., Dlouhý A., Somsen C., Bei H., Eggeler G., George E.P. (2013). The influences of temperature and microstructure on the tensile properties of a CoCrFeMnNi high–entropy alloy. Acta Mater..

[9] Tang Y., Wang R., Xiao B., Zhang Z., Li S., Qiao J., Bai S., Zhang Y., Liaw P.K. (2023). A review on the dynamic–mechanical behaviors of high–entropy alloys. Prog. Mater. Sci..

[10] Wang H., Koyama M., Hojo T., Akiyama E. (2021). Hydrogen embrittlement and associated surface crack growth in fine–grained equiatomic CoCrFeMnNi high–entropy alloys with different annealing temperatures evaluated by tensile testing under in situ hydrogen charging. Int. J. Hydrogen Energy.

[11] Fu G., Liu X., Yi X., Zhang S., Cao X., Meng X., Gao Z., Wang H. (2023). Development of High–Entropy Shape–Memory Alloys: A Review. Metals.

[12] Yang T., Zhao Y.L., Tong Y., Jiao Z.B., Wei J., Cai J.X., Han X.D., Chen D., Hu A., Kai J.J. (2018). Multicomponent intermetallic nanoparticles and superb mechanical behaviors of complex alloys. Science.

[13] Hamdi H., Abedi H.R., Zhang Y. (2023). A review study on thermal stability of high entropy alloys: Normal/abnormal resistance of grain growth. J. Alloys Compd..

[14] Hua D., Xia Q., Wang W., Zhou Q., Li S., Qian D., Shi J., Wang H. (2021). Atomistic insights into the deformation mechanism of a CoCrNi medium entropy alloy under nanoindentation. Int. J. Plast..

[15] Xiong W., Guo A.X., Zhan S., Liu C.-T., Cao S.C. (2022). Refractory high–entropy alloys: A focused review of preparation methods and properties. J. Mater. Sci. Technol..

[16] Yan X.H., Li J.S., Zhang W.R., Zhang Y. (2018). A brief review of high–entropy films. Mater. Chem. Phys..

[17] Zhang Y.Q., Wang D.D., Wang S.Y. (2022). High–entropy alloys for electrocatalysis: Design, characterization and applications. Small.

[18] Güler S., Alkan E.D., Alkan M. (2022). Vacuum arc melted and heat treated AlCoCrFeNiTiX based high–entropy alloys: Thermodynamic and microstructural investigations. J. Alloys Compd..

[19] Lu Y., Dong Y., Jiang H., Wang Z., Cao Z., Guo S., Wang T., Li T., Liaw P.K. (2020). Promising properties and future trend of eutectic high entropy alloys. Scr. Mater..

[20] Braic V., Balaceanu M., Braic M., Vladescu A., Panseri S., Russo A. (2012). Characterization of multi–principal–element (TiZrNbHfTa)N and (TiZrNbHfTa)C coatings for biomedical applications. J. Mech. Behav. Biomed. Mater..

[21] Nagase T., Rack P.D., Noh J.H., Egami T. (2015). In–situ TEM observation of structural changes in nano–crystalline CoCrCuFeNi multicomponent high–entropy alloy (HEA) under fast electron irradiation by high voltage electron microscopy (HVEM). Intermetallics.

[22] Buravlev I., Shichalin O., Marmaza P., Kolodeznikov E., Dvornik M., Sakhnevich A., Buravleva A., Chuklinov S., Papynov E. (2024). Microstructural evolution and mechanical behavior of WC–4wt.%TiC–3wt.%TaC–12wt.%Co refractory cermet consolidated by spark plasma sintering of mechanically activated powder mixtures. Adv. Powder Technol..

[23] Simonenko E.P., Simonenko N.P., Papynov E.K., Shichalin O.O., Belov A.A., Nagornov I.A., Gorobtsov P.Y., Kuznetsov N.T. (2022). Effect of nanocrystalline SiC addition on reactive SPS and oxidation resistance of Ta4HfC5 ceramics. Ceram. Int..

[24] Shichalin O., Buravlev I., Papynov E., Buravleva A., Sakhnevich V., Dvornik M., Vlasova N., Gerasimenko A., Reva V., Yudakov A. (2022). Comparative study of WC–based hard alloys fabrication via spark plasma sintering using Co, Fe, Ni, Cr, and Ti binders. Int. J. Refract. Met. Hard Mater..

[25] Zeraati M., Khayati G.R., Feizabad M.H.K. (2024). Microstructure and Magnetic Characteristics of AlCrCuFeNiTi High–Entropy Alloy Prepared by Mechanical Alloying Followed by Spark Plasma Sintering. J. Mater. Eng. Perform..

[26] Adabavazeh Z., Hosseini S., Karimzadeh F., Abbasi M. (2024). Fabrication and performance assessment of CuFeNiMnTi high entropy alloy through mechanical alloying and spark plasma sintering. Mater. Today Commun..

[27] Yurchenko N., Shaysultanov D., Povolyaeva E., Moskovskikh D., Zherebtsov S., Stepanov N. (2024). Structure and mechanical properties of low–density AlCrFeTiX (X = Co, Ni, Cu) high–entropy alloys produced by spark plasma sintering. J. Alloys Compd..

[28] Chen Y.-T., Chang Y.-J., Murakami H., Gorsse S., Yeh A.-C. (2020). Designing high entropy superalloys for elevated temperature application. Scr. Mater..

[29] Chen D., He F., Han B., Wu Q., Tong Y., Zhao Y., Wang Z., Wang J., Kai J.-J. (2019). Synergistic effect of Ti and Al on L12–phase design in CoCrFeNi–based high entropy alloys. Intermetallics.

[30] Zhao L., Jiang L., Yang L., Zhang W., Ji G., Zhou X., Curtin W., Chen X., Liaw P., Chen S. (2022). High throughput synthesis enabled exploration of CoCrFeNi–based high entropy alloys. J. Mater. Sci. Technol..

[31] Zhi Q., Tan X., Liu Z., Liu Y., Zhang Q., Chen Y., Li M. (2021). Effect of Zr content on microstructure and mechanical properties of lightweight Al_2_NbTi_3_V_2_Zr_x_ high entropy alloy. Micron.

